# Structured Large Language Model Workflows for Motivational Interviewing in Health Behavior Change: Proof-of-Concept Study

**DOI:** 10.2196/94036

**Published:** 2026-07-06

**Authors:** Akshaye Shenoi, Tianze Li, Ahmad Ishqi Jabir, Amanda Pitkethly, Elgar Fleisch, Tobias Kowatsch, Jacqueline Louise Mair

**Affiliations:** 1 Future Health Technologies Programme, Singapore-ETH Centre, Campus for Research Excellence and Technological Enterprise (CREATE), 1 Create Way138602, Singapore, +65 6684 2900; 2 Centre for Digital Health Interventions, Department of Management, Technology, and Economics, ETH Zurich, Zurich, Switzerland; 3 School of Applied Sciences, Edinburgh Napier University, Edinburgh, Scotland, United Kingdom; 4 Centre for Digital Health Interventions, Institute of Technology Management, University of St. Gallen, St. Gallen, Switzerland; 5 Institute for Implementation Science in Health Care, University of Zurich, Zurich, Switzerland; 6 School of Medicine, University of St. Gallen, St. Gallen, Switzerland

**Keywords:** artificial intelligence, behavioral health, health coaching, digital health, mHealth, conversational agents, large language models, motivational interviewing, mobile health

## Abstract

**Background:**

Motivational interviewing (MI) is an effective approach for supporting health behaviorchange, but face-to-face delivery is resource-intensive and difficult to scale. Rule-based conversational agents (CAs) can improve access; however, their scripted interactions and limited language flexibility constrain MI delivery. While large language models (LLMs) are increasingly being used for MI coaching, their conversational fidelity and quality compared with human coaches and rule-based CAs remain understudied.

**Objective:**

This study aimed to describe the development of an LLM-based CA, Artificially Intelligent Motivational Interviewing (Aimi), orchestrated with structured workflows, and to evaluate its feasibility, conversational fidelity, and user perceptions during MI coaching interactions.

**Methods:**

We developed Aimi using structured LLM workflows designed to enhance MI fidelity. We conducted a within-participants study, where 18 adults interacted with (1) Aimi, (2) a novice MI-trained human coach, and (3) a rule-based CA during live text-based role-play coaching sessions. Transcripts were independently evaluated by an MI expert using the Motivational Interviewing Skill Code, Version 2.0 (MISC-2), to assess MI competency and fidelity. Participants completed a user experience questionnaire to provide general feedback and to assess session alliance, dialogue relevance, empathy, engagement, linguistic quality, and perceived motivation to change. Feedback from users was thematically summarized and categorized under strengths and weaknesses for each approach.

**Results:**

Aimi achieved fidelity scores comparable to those of the novice human coach and higher than those of the rule-based CA on summary metrics, including higher reflection-to-question ratios (median 0.84, IQR 0.62-0.92 vs 0.62, IQR 0.42-0.74 vs 0.25, IQR 0.17-0.38), more complex reflections (median 66.67%, IQR 46.97%-76.92% vs 50%, IQR 34.38%-61.88% vs 0.00%, IQR 0%-50%), and greater elicitation of client change talk (median 90.83%, IQR 85.89%-100% vs 73.21%, IQR 63.10%-83.19% vs 66.67%, IQR 57.86%-81.94%). User experience ratings showed no significant differences across conditions. User feedback revealed distinct strengths and limitations across the coaching interactions. Participants described Aimi’s interactions as personalized, fluid, and adaptive, though sometimes overly reflective and lengthy. The novice human coach was viewed as empathetic and supportive but slow to respond, whereas the rule-based coach was viewed as efficient and structured yet limited in depth and personalization.

**Conclusions:**

This study demonstrates the technical feasibility of structured LLM-workflows for MI coaching and their capacity to maintain conversational fidelity comparable to that of a novice MI-trained human coach. Given the role-play paradigm, single-rater coding, and small convenience sample, these comparative findings should be interpreted as exploratory. Our findings serve as a foundational baseline for the development of scalable behavior change interventions in clinical settings.

## Introduction

Noncommunicable diseases (NCDs), including cardiovascular disease, diabetes, and mental health disorders, are the leading cause of death and disability worldwide [1,2]. Many of the underlying risk factors for NCDs, such as high blood pressure, elevated BMI, and high blood glucose, are modifiable through lifestyle behavior change [3-5]. While improvements in physical activity, diet, sleep, and stress management have been shown to significantly reduce the risk of NCD onset and progression [6-8], implementing and maintaining long-term behavior change remains a major public health challenge.

Motivational interviewing (MI) is a collaborative, goal-oriented style of communication used to strengthen an individual’s motivation for and commitment to behavior change [9]. Based on the principles of empathy, autonomy, and evocation, MI has demonstrated effectiveness across a range of health behaviors including physical activity, smoking, alcohol, and substance use disorders [10,11]. Traditionally delivered by trained practitioners in face-to-face settings, MI requires nuanced conversational skills, such as affirming autonomy, reflective listening, and the ability to elicit and respond to change talk. While effective, delivering MI at scale remains a challenge due to the time, cost, and training requirements involved [12].

In recent years, conversational agents (CAs) have emerged as a promising avenue to extend the reach of health coaching interventions such as MI [13,14]. Rule-based CAs, built on scripted dialogue trees, have been used to deliver health literacy support [15,16] and evidence-based strategies such as MI [17,18] for a range of health care challenges [19]. While these CAs are more scalable than traditional approaches and offer some degree of structure and safety, their inherent inability to support linguistically complex MI dialogue (eg, complex reflections, summarizing, and so on) limits their ability to adapt meaningfully to an individual’s evolving context, motivation, and needs [20].

With the emergence of large language models (LLMs), there is a growing opportunity to develop more sophisticated automated CAs that can emulate the conversational flow and therapeutic alliance found in human-delivered MI. For example, recent research supports the potential for LLMs to deliver MI for health behaviors such as alcohol use [21,22], smoking cessation [23-25], and lifestyle improvement [26-28]. Despite rising interest in LLM-based MI systems, empirical evaluations remain limited. Few studies have explored the conversational quality, fidelity, and user perceptions of LLM-based MI coaching alongside current rule-based systems and human coaches on real users. Most rely on LLM-simulated users—a method that is known to overstate rational behavior in humans [29]—or single-condition designs that do not include human coaches. Furthermore, studies often use single-prompt approaches to deliver MI sessions [22,24,27,30]. While straightforward, the performance of LLMs is known to degrade as the length of a multiturn conversation increases, often leading to conversational drift [31]; in contrast, structured workflows can help alleviate these issues by guiding the model through more constrained and focused interactions. As such, there is a need to explore whether LLM-based CAs can successfully deliver MI interactions that are not only linguistically fluent but also therapeutically aligned and safe [32].

This study presents a proof-of-concept evaluation of Artificially Intelligent Motivational Interviewing (Aimi), an LLM-based CA that operationalizes MI strategies through a structured workflow. Our primary objective is to evaluate the technical feasibility and conversational fidelity of Aimi, using a text-based novice human coach and a rule-based CA as directional benchmarks to contextualize performance. While Aimi is designed to be applicable across health behaviors, we focus on physical activity coaching as a controlled test case. Specifically, the study addresses the following research questions (RQs): RQ1: To what extent does MI fidelity vary across Aimi, a novice human coach, and a rule-based CA? and RQ2: How do users perceive the linguistic quality, usability, and relevance of the dialogue across 3 approaches?

## Methods

In this section, we provide an overview of MI and describe the system design and architecture of Aimi. We then describe the study protocol and evaluation metrics used to measure the effectiveness of Aimi.

### Overview of MI

At the heart of MI lies the “spirit”—Partnership, Acceptance, Compassion, and Empowerment—which underpins all interactions. The interviewer must not assume an authoritative role but instead act as a collaborative partner drawing out the client’s own reasons for change. A session typically advances in the following four stages associated with distinct objectives that guide the interview:

- Engaging: establishing a trusting and respectful therapeutic relationship. The interviewer can, for example, use the open-ended questions, affirmations, reflective listening, and summarizing (OARS) strategy to establish rapport.
- Focusing: centers on collaboratively identifying a specific direction for change.
- Evoking: aims to elicit the client’s intrinsic motivation for change. The interviewer reflects and reinforces two types of change-talk: preparatory language, which expresses Desire, Ability, Reasons, and Need, and implementing language, which includes Commitment, Activation, and Taking Steps.
- Planning: transitions to actionable steps by helping the client articulate goals.

A key feature of MI is the distinction between a client’s change-talk and sustain-talk language, that is, client language that is oriented toward making a behavioral change or maintaining current behavior (the status quo). MI seeks to elicit and selectively reinforce change-talk using a variety of strategies, including the use of evocative questions and reflections. On the other hand, sustain-talk is not reinforced, but the conversation is gently guided back toward exploring the gap between the client’s current behavior and their goals, ultimately eliciting change-talk.

### Aimi: System Design

Aimi was codeveloped by a team of psychologists, behavior change experts, and computer scientists to deliver MI-consistent dialogue in a scalable manner. Aimi emulates the core processes of MI—engaging, focusing, evoking, and planning—and is built to maintain adaptability across diverse behavioral domains, ensuring fidelity to MI principles while allowing personalization for individual user needs.

The following principles guided the development:

- High fidelity to MI: the system prioritizes strong adherence to MI principles and conversational techniques that enhance intrinsic motivation and support an individual’s commitment to behavior change.
- Personalization: the system adapts dynamically to the unique needs, preferences, and conversational cues of each user. This allows the agent to tailor responses in real time as the user’s circumstances, readiness, and goals evolve across the session.
- Intervention agnostic: the system is designed to be applicable across a wide range of health behaviors and intervention goals (eg, increasing physical activity, improving diet, and managing stress), without being restricted to a specific condition or behavior domain. This makes Aimi suitable for integration into various digital health programs.

**Figure 1.**
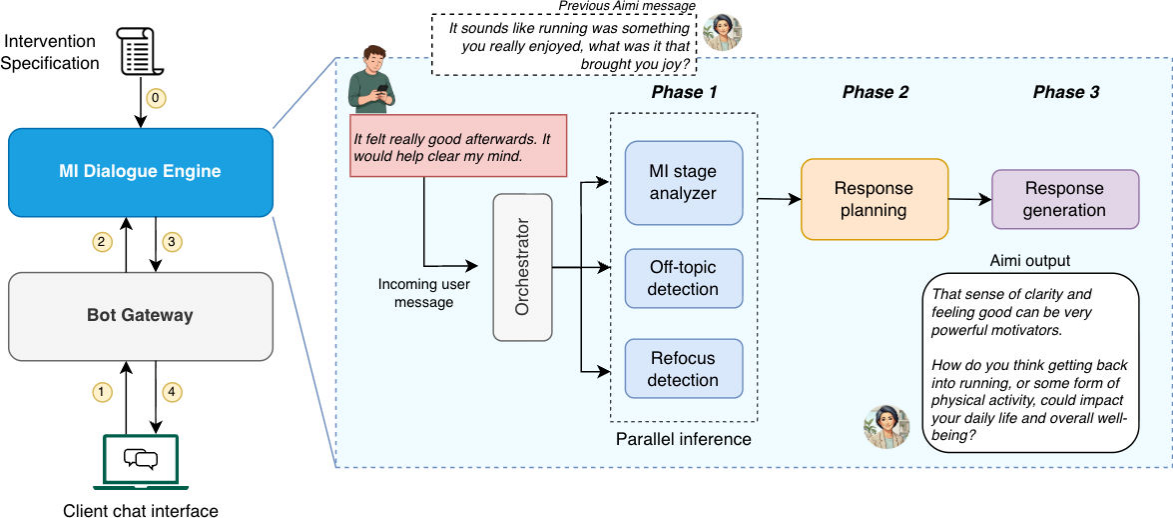
High-level system overview of Aimi. It comprises an Intervention Specification that defines key components of the target intervention (eg, target users, guidelines, and so on), a Bot Gateway for handling client chat interface communication, and the core MI Dialogue Engine, which orchestrates large language model workflows to guide the conversation with a client.

At a high level, Aimi consists of three core components illustrated in Figure 1. First, an intervention specification systematically defines key components of the target intervention to tailor the MI session for a specific use case. Second, the MI Dialogue Engine, consisting of a carefully designed LLM workflow, guides the conversation with a client in an MI-consistent fashion. Finally, a Bot Gateway manages message input and output with the client.

### Intervention Specification

To configure an intervention, appropriate context must be clearly provided to Aimi. The intervention specification is a structured text document that defines the intervention’s target behavior, target user, intervention-specific guidelines or prescriptions, and intervention schedule. Thus, the intervention specification forms the “blueprint” of all Aimi interactions with the users.

We use markdown format for this document. An example intervention specification is provided in Multimedia Appendix 1.

### MI Dialogue Engine

#### Overview

The MI Dialogue Engine contains the core logic that translates MI principles and strategies into a natural language capable CA. The engine can be configured to use any off-the-shelf foundation models, for example, OpenAI’s GPT-4.5 [33] or Meta’s Llama 3 [34]. To generate coherent and therapeutically aligned dialogue, we augment the foundational model using a combination of techniques such as workflow-routing and prompt-chaining [35].

Specifically, the Dialogue Engine is composed of an orchestrator that routes every message through the workflow described in Figure 1. A node on the workflow is an atomic task that is carefully mapped to an MI concept and consists of a prompt and an invocation to the LLM. For instance, the Off-topic Detection task invokes the LLM with a prompt instructing the model to detect off-topic client utterances. The orchestrator then evaluates a condition (or a set of conditions) on the output to route the message to the next appropriate task in the workflow. In this way, outputs are chained together to produce safe and MI-consistent responses.

To effectively emulate an MI session, the workflow is designed to guide the conversation along the four stages of MI, that is, engaging, focusing, evoking, and planning. This is achieved sequentially in the following three phases: (1) identifying whether the goals within the current MI stage are met and advancing the stage if appropriate, (2) applying stage-specific MI strategies to draft a response strategy, and (3) crafting the actual response. The tasks within each phase are as follows:

#### Phase 1

The principal objective in this phase is to determine the current MI stage. At the beginning of each session, the MI stage is set to engaging. As the conversation advances, the Stage Analyzer task ascertains if the goals associated with the current MI stage have been achieved. These goals, derived from the MI handbook [9], are provided to the task as summarized JSON strings. Once the goals are met, the orchestrator advances the MI stage sequentially (engaging → focusing → evoking → planning). The resulting MI stage is subsequently used by downstream tasks in Phases 2 and 3.

Simultaneously, 2 other auxiliary tasks also monitor client utterances to ensure the conversation remains aligned to the broader intervention objectives. The off-topic detection task identifies if the client’s message potentially diverges from the focus—for instance, expressing job stress or work dissatisfaction in a session aimed at improved physical activity. If detected, a special off-topic strategy is applied in the subsequent phase; the client’s message is acknowledged, but an attempt is made to gently steer back to the primary topic. On the other hand, the Refocus Detection task identifies if a client appears to redefine their goals in the planning stage of MI. For example, expressing doubt about completing an existing goal and indicating interest in taking a different direction. In this case, the MI stage is rolled back to evoking, and an attempt is made to reinforce commitment and planning toward the new goal.

#### Phase 2

Once the MI stage has been reliably detected, the workflow enters the Response Planning phase. Here, a context-appropriate response plan is generated based on (1) the current MI stage, (2) the intervention specification, and (3) the conversation history with the client. It consists of a single task that provides the LLM with a list of MI strategies appropriate for the current MI stage. For instance, if the current stage is Engaging, the system selects stage-appropriate techniques such as OARS (along with other stage-specific techniques) to generate an appropriate response plan.

#### Phase 3

Finally, the generated plan is consumed by the Response Generation task to finally produce the actual response. This pattern—where the response planning is decomposed into an intermediate step—has been shown to produce higher quality outputs and enhance adherence to MI principles [26]. The MI Dialogue Engine passes this generated response to the Bot Gateway, which eventually delivers it to the client. All prompts used to guide the model are provided in Multimedia Appendix 2.

### Bot Gateway and Chat Interface

The Bot Gateway is a simple web-based service that manages client input and output messages. It also manages the conversation history for each client along with key variables such as client name, user ID, and current MI stage. These variables are used by the Dialogue Engine while executing the workflow.

**Figure 2.**
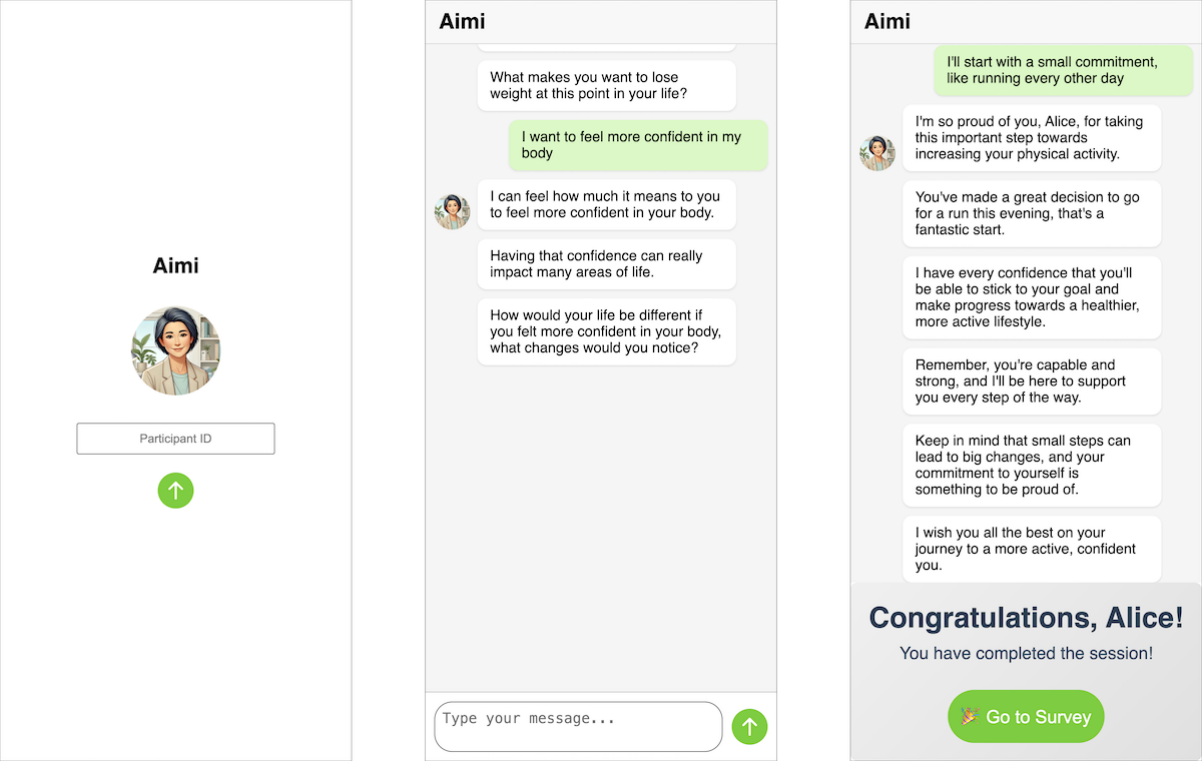
Mobile-based chat interface for interacting with Aimi.

A simple chat interface allows the client to interact with the service from a web browser or a mobile app, as shown in Figure 2.

### Implementation

We implemented the Dialogue Engine using the Python programming language and deployed it using the Llama 3.3-70B model hosted on Amazon Web Services Bedrock as the foundation language model. Model selection was guided by the need to balance response quality with response latency. During prototyping, we observed that using a medium-sized model achieved sufficiently coherent linguistic quality while also maintaining an optimal response time. Furthermore, Llama 3.3 is an open source model that allowed us to retain full control over data flows and operate the service in a regulatory-compliant manner.

The Bot Gateway was implemented using Microsoft Bot Framework [36], which provides key features such as maintaining parallel user conversations and persisting them to storage.

### Proof-of-Concept Study

#### Overview

We conducted a foundational study to assess the performance of Aimi, in terms of its fidelity to MI counseling (RQ1) and users’ perceived interaction quality (RQ2), when compared against human and rule-based coaching approaches. We focused on physical activity as a controlled test case.

#### Study Design

We conducted a mixed methods human evaluation study between February and March 2025 using a within-participants design to explore the performance of the following three types of MI coaching approaches:

1. Aimi: our LLM-based CA designed to deliver MI.
2. Novice MI-trained human coach: a member of the research group undertook a 10-hour online course and a 2-day in-person refresher with expert MI trainers (members of the Motivational Interviewing Network of Trainers). The training included an overview of MI theory and strategies, role-playing, and MI skill evaluation tailored to the context of digital intervention delivery. Expert MI trainers require extensive certifications and years of clinical practice, making such resources inaccessible for large-scale deployment. Therefore, our choice of a novice human coach with foundational training was motivated by the need for a more scalable and pragmatic approach that could be compared with automated systems. The session was delivered via a simple web-based chat interface.
3. Rule-based MI-inspired CA: a predefined evidence-based dialogue script was developed using MI and physical activity behavior change strategies. This script has been used and evaluated in previous studies [17,18]. Note that, unlike Aimi, the rule-based CA does not possess natural language processing capabilities. Thus, participants interacted using predefined answer options on a web-based chat interface.

#### Participants

We recruited a convenience sample of healthy adults aged 21‐59 years who were fluent in English (Table 1). Participants were located in Singapore and were recruited through study advertisements on the research center’s internal chat forum and mailing lists.

**Table 1.** Participant characteristics.

Characteristic and option	Count, n (%)
Sex	
Female	11 (61.1)
Male	7 (38.9)
Age (years)	
21‐30	9 (50.0)
31‐40	9 (50.0)
>41	0 (0.0)
Education level	
Postgraduate (eg, master’s degree, PhD)	10 (55.6)
Undergraduate (eg, bachelor’s degree)	6 (33.3)
College (eg, diploma, certificate)	2 (11.1)
Adherence to physical activity guidelines (≥150 min during the past 7 d)	
Yes	12 (66.7)
No	6 (33.3)
Average daily step count (self-reported)	
0‐5000	3 (16.7)
5001‐10,000	11 (61.1)
>10,000	1 (5.6)
Unknown	3 (16.7)
Familiarity with chatbots	
Extremely familiar	5 (27.8)
Moderately familiar	6 (33.3)
Somewhat familiar	5 (27.8)
Slightly familiar	2 (11.1)
Used chatbots before	
Yes	16 (88.9)
No and never considered	1 (5.6)
No, but considered	1 (5.6)
Chatbot use frequency	
Every day	1 (5.6)
Almost every day	8 (44.4)
Sometimes	5 (27.8)
Rarely	2 (11.1)
Unknown	2 (11.1)

#### Procedure

Participants attended a 1.5-hour in-person, laboratory-based study session. After receiving a verbal explanation of the study procedures and providing written informed consent, each participant was provided with a laptop and an instructional leaflet. The leaflet outlined (1) how to access the coaching sessions, (2) the order in which to complete them, and (3) the steps for submitting postsession feedback. Each session was assigned a unique identifier to link the conversation transcript with the corresponding postsession survey. Due to logistical constraints and longer response times associated with the novice human coach, true probabilistic randomization was not feasible. Instead, a quasi-randomized approach was used whereby participants completed the 3 coaching sessions in a predefined sequence dictated by operational availability. While the specific sequence allocation was blinded to the participants, the conditions were not fully counterbalanced across all possible orderings.

Participants were instructed to assume the role of a physically inactive individual to provide a consistent baseline across the approaches. When ready, participants activated a coaching session through a web-based chat interface accessed via a laptop, using a unique ID that was linked to the allocated condition for randomization and tracking. They then engaged in a text-based conversation with one of the three MI coaches—Aimi, a human coach, or a rule-based coach—without being informed which coach they were interacting. However, even though blinding was attempted, it could not be strictly enforced because of the inherent differences in the technical characteristics of the conditions (eg, response latencies, predefined responses, and so on). Each coaching session lasted approximately 20 minutes, depending on the conversational flow. At the end of the interaction, participants completed a short survey evaluating (1) working alliance with the coach, (2) dialogue relevance, (3) linguistic quality, (4) empathy, (5) engagement, (6) usability, (7) motivation to change, and (8) open-ended feedback on the experience. This process was repeated until participants had completed all 3 coaching sessions and associated evaluations.

At the end of the study, each participant was reimbursed with an e-voucher valued at SGD 30 (approximately US $23).

### Measures

#### Participant Demographics

Upon first completing the short survey, participants were asked to report demographic characteristics, including gender, age, nationality, and education level, as well as their physical activity level and prior experience using chatbots.

#### Expert-Rated MI Fidelity

The quality and fidelity to MI were assessed by an expert member of the Motivational Interviewing Network of Trainers using the Manual for the Motivational Interviewing Skill Code Version 2.0 (MISC-2) [37]. Specifically, each conversational turn in the transcripts was coded in relation to the behavior goal. The coding system produced the following three scores: Behavior Counts, Summary Scores, and Global Ratings.

Behavior Counts capture specific coach and client behaviors through sequential coding, independent of global ratings. There are 15 major categories of coach behavior in MISC-2: advise with and without permission, affirm, confront, direct, emphasize control, facilitate, filler, giving information, open and closed questions, raise concern with and without permission, simple and complex reflection, reframe, support, structure, and warn. Counts are determined through coding and tallying the frequency of specific language behaviors using 19 coach codes and 7 client codes. Client statements are attributed either a positive or negative valence that reflects language (ie, reason, desire, ability, need, commitment, and taking steps) toward or away from the behavior goal (change or sustain talk).

Summary Scores are calculated from behavior count frequencies to provide concise indicators of MI competence and session dynamics. These include ratios (eg, reflections-to-questions ratio and open-to-closed questions ratio) and percentages (eg, percent complex reflections and percent MI-consistent behaviors) that quantify the coach’s adherence to MI principles and the client’s engagement in change language.

Global Ratings are based on an overall holistic impression of the entire coach-client interaction. Ratings consider three global coach dimensions—acceptance (unconditional positive regard), empathy (understanding the client’s perspective), and MI spirit (encompassing collaboration, evocation, and autonomy support)—and 1 client dimension (self-exploration). All ratings use a 7-point Likert scale (1-7), with coders beginning at a baseline score of 4 and adjusting upward or downward based on the coach’s or client’s performance throughout the session. Higher scores indicate higher quality.

#### Perceived User Experience

In the absence of a validated evaluation tool for human-LLM interactions, we used questionnaire items from previous research studies evaluating CAs [38]. We list the items below and provide the full questionnaire in Multimedia Appendix 3.

- Session Alliance Inventory: measures the collaborative and affective bond between the coach and the user [39].
- Dialogue Relevance: assesses how focused, purposeful, and on-topic the conversation was during the session.
- Linguistic Quality: captures the fluency, coherence, and naturalness of the conversation.
- Empathy: reflects the perceived empathy of the coach’s conversations and their ability to understand and resonate with the participant’s feelings.
- Engagement: indicates how engaging conversation was for the participant.
- Usability: evaluates the clarity and interpretability of the coach’s communication.
- Perceived Motivation: assesses the coach’s role in fostering motivation and readiness to change behavior.
- Open-Ended Feedback: captures additional insights with 2 open-ended questions: (1) What have you enjoyed most or least about this coaching session? (2) What could be improved about this coaching session?

Session alliance was measured on a 6-point Likert scale anchored from 1 (“not at all”) to 6 (“completely”), while the remaining outcomes were measured on a 5-point Likert scale anchored from 1 (“strongly disagree”) to 5 (“strongly agree”).

#### Message Response Latency

Response latency was defined as the elapsed time between a user input and the corresponding coach response and was computed for all sessions. Latency distributions were summarized using median, mean, SD, and percentile-based thresholds.

### Data Analysis

No a priori power calculation was performed. The sample size of 18 was determined pragmatically based on the formative nature of the study and is consistent with conventions for feasibility and proof-of-concept studies [40]. Comparative inferential statistics are reported as exploratory and effect-size oriented rather than confirmatory.

To explore differences in user experience and fidelity to MI between coaching types, we first checked the data for normality using the Shapiro-Wilk test. Because the data were not normally distributed, we used the Friedman test to compare participant ratings across the 3 coaching conditions. Post hoc pairwise comparisons were conducted using Wilcoxon signed-rank tests with Holm-Bonferroni correction.

### Open-Ended Feedback

One member of the research team reviewed the open-ended responses from the questionnaire and coded the data. The data were reviewed again and codes were refined before being grouped into themes. Themes were then categorized as strengths (aspects participants liked most) and weaknesses (aspects participants liked least or suggested for improvement) for each coach. Quotes are used to contextualize the findings.

### Ethical Considerations

The study was approved by the Institutional Review Board of the National University of Singapore (approval NUS-IRB-2025‐98). Informed consent (Multimedia Appendix 4) was obtained from all participants before enrollment. Participants were informed that their responses would be kept confidential, with data anonymized and securely stored. Access to the data was restricted to the research team, and all identifying information was removed before analysis.

## Results

### Participant Demographics

We recruited 18 participants for this study. Table 1 provides the participant characteristics, including demographic profile, current physical activity level, and prior experience using chatbots. Notably, most participants reported high or moderate familiarity with chatbots, and a majority of them (n=16, 88.9%) had used them before. Half of the participants (n=9, 50%) reported that they used chatbots every day or almost every day. Although the inclusion criterion permitted ages 21‐59 years, the recruited convenience sample skewed young, with all participants aged 21‐40 years.

### Expert-Rated MI Fidelity

Table 2 compares the MISC-2 summary and global scores for each coaching condition. The data suggest that across summary metrics, Aimi achieved higher scores than both the novice human coach and the rule-based CA. Notably, Aimi appeared to elicit client change-talk in a greater proportion of utterances (median 90.83%, IQR 85.89%‐100%) compared with the human coach (median 73.21%, IQR 63.10%‐83.19%) and the rule-based CA (median 66.67%, IQR 57.86%‐81.94%). Aimi also showed a higher reflection-to-question ratio and used a higher proportion of complex reflections: median 66.67% (IQR 46.97%‐76.92%) compared to median 50% (IQR 34.38%‐61.88%) and 0% (IQR 0%‐50%) for the human and rule-based CA. All coaching conditions demonstrated high levels of MI-consistent behavior (median 100%, IQR >95%).

**Table 2.** MISC-2^a^ summary and global scores (N=18) for MI^b^ fidelity across each coach.

Measure	Aimi, median (IQR)	Novice human, median (IQR)	Rule-based, median (IQR)	*P* value^c^	Pairwise *P* value (A-H)^d^	Pairwise *P* value (H-R)^d^	Pairwise *P* value (A-R)^d^
MISC-2^a^ summary scores							
Ratio reflections-to-questions	0.84 (0.62‐0.92)	0.62 (0.42‐0.74)	0.25 (0.17‐0.38)	.005^e^	.83	.003^e^	.003^e^
Open questions (%)	89.57 (83.65‐93.33)	61.25 (57.14‐73.75)	50.00 (38.12‐50.00)	.001^e^	.02^e^	.31	.001^e^
Complex reflections (%)	66.67 (46.97‐76.92)	50.00 (34.38‐61.88)	0.00 (0.00‐50.00)	.02^e^	.75	.33	.03^e^
MI^b^ consistent (%)	100 (96.58‐100)	100 (95.66‐100)	100 (100‐100)	.08	.99	.43	.33
Client change talk (%)	90.83 (85.89‐100)	73.21 (63.10‐83.19)	66.67 (57.86‐81.94)	.004^e^	.001^e^	.99	.04^e^
MISC-2 global scores							
Overall coach rating	4.00 (4.00‐4.00)	4.00 (4.00‐5.00)	3.50 (3.00‐4.00)	.002^e^	.75	.01^e^	.11
Acceptance	6.00 (6.00‐6.00)	6.00 (5.00‐6.00)	5.00 (5.00‐5.38)	.02^e^	.99	.74	.02^e^
Empathy	5.00 (5.00‐5.00)	5.00 (5.00‐5.00)	4.00 (4.00‐5.00)	.01^e^	.99	.07	.06
MI spirit	5.00 (5.00‐6.00)	5.00 (5.00‐5.00)	4.25 (4.00‐5.00)	.06	.99	.57	.24
Client self-exploration	4.00 (3.12‐4.00)	4.00 (3.25‐4.00)	3.00 (3.00‐3.38)	.02^e^	.99	.04^e^	.11

a MISC-2: Motivational Interviewing Skill Code Version 2.0.

b MI: motivational interviewing.

c *P* values for the Friedman omnibus test.

d *P* values for pairwise comparisons using the Wilcoxon signed-rank test with Holm-Bonferroni correction. A, H, and R represent Aimi, human coach, and rule-based conversational agent, respectively.

e "*” indicates statistical significance at *P*<.05.

Global ratings were similar for Aimi and the novice human coach, with both achieving an overall median coach rating of 4, corresponding to average MI competence. The median and ranges of scores were also similar across acceptance, empathy, MI spirit, and client self-exploration. In contrast, the rule-based CA scored lower by 0.5 to 1 point across these measures.

### Perceived User Experience

Table 3 summarizes participants’ perceived user experience across the 3 coaching conditions. A small fraction of survey responses contained missing data (5/1350 total responses, 0.37%), which were mean-imputed based on the other responses for that coach in the respective questionnaire. Overall, the omnibus test indicated no statistically significant differences in user experience ratings across conditions.

**Table 3.** Perceived user experience ratings across each coach.

Measures	Aimi, median (IQR)	Novice human, median (IQR)	Rule-based, median (IQR)	*P* value^a^	Pairwise *P* value (A-H)^b^	Pairwise *P* value (H-R)^b^	Pairwise *P* value (A-R)^b^
Session alliance	4.25 (3.88‐4.62)	4.17 (3.54‐4.75)	3.83 (2.75‐4.25)	.11	.99	.04^c^	.99
Perceived motivation	3.88 (3.06‐4.00)	3.50 (2.75‐4.25)	3.00 (2.62‐3.50)	.99	.99	.999	.99
Linguistic quality	3.58 (2.81‐4.00)	4.00 (3.06‐4.69)	3.12 (2.56‐3.50)	.99	.99	.99	.99
Dialogue relevance	3.83 (3.08‐4.25)	4.00 (3.42‐4.25)	4.00 (3.42‐4.00)	.99	.99	.99	.99
Empathy	4.00 (3.12‐4.00)	4.00 (3.62‐4.00)	3.25 (2.62‐4.00)	.77	.99	.35	.99
Engagement	4.00 (2.00‐4.00)	3.50 (3.00‐4.00)	3.00 (3.00‐4.00)	.99	.99	.99	.99
Usability	3.75 (3.31‐4.19)	4.00 (3.50‐4.62)	3.75 (3.25‐4.00)	.99	.99	.89	.99

a *P* values for Friedman omnibus test.

b *P* values for pairwise comparisons using the Wilcoxon signed-rank test with Holm-Bonferroni correction. A, H, and R represent Aimi, human coach, and rule-based conversational agent, respectively.

c * indicates statistical significance at *P*<.05.

### Message Response Latency

**Figure 3.**
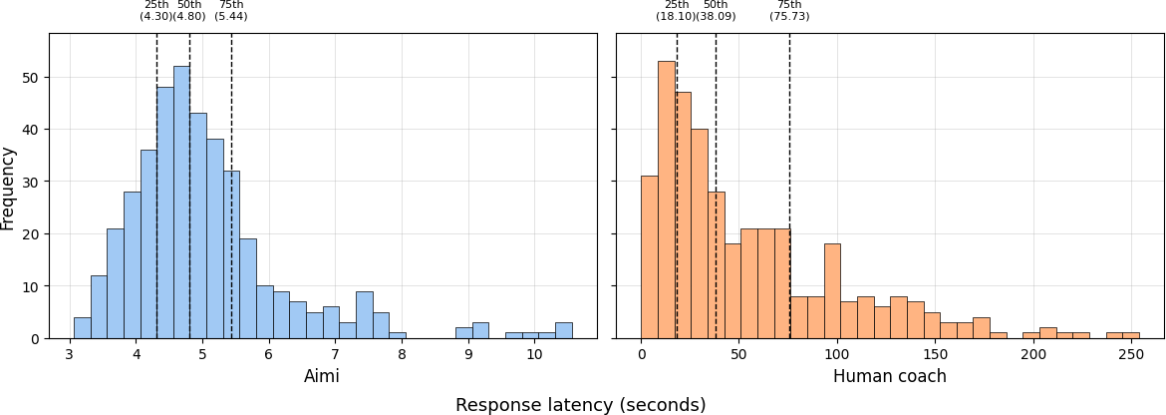
Distribution of response latency (in seconds) for Aimi and the novice human coach. Vertical dotted lines mark the 25th, 50th, and 75th percentile values. The rule-based conversational agent provided instantaneous responses with negligible delay.

Figure 3 provides the distribution of Aimi and the novice human coach’s response latencies across the sessions. Due to a technical error with application logging, we were unable to compute the response latencies for the first Aimi session. The error did not affect any other analysis. We thus report the data for the remaining 17 sessions. The median latency per response was approximately 4.8 (IQR 4.3-5.4) seconds for Aimi, with 90% of all responses delivered within 6.5 seconds. The mean latency was 5.1 (SD 1.23) seconds.

In contrast, the novice human coach responded with higher latency and variation (median 38.09 seconds, IQR 18.1-75.73 seconds; mean 54.9 seconds, SD 48.2 seconds). The rule-based CA provided instantaneous responses with negligible delay because it did not require natural language processing.

### Open-Ended Feedback

#### Overview

Participants provided contextual insights into the strengths and weaknesses of each coaching interaction, which are summarized in Table 4.

**Table 4.** Participant-reported strengths and weaknesses of each coaching interaction.

Coach	Strengths	Weaknesses
Aimi	Contextual understanding Fluid and natural dialogue Adaptive and responsive interactions Ability to evoke motivation and readiness for action	Overuse of reflections and summaries Redundant paraphrasing Lack of actionable guidance
Novice human coach	Emotional attunement Adaptive and responsive interactions Authentic human connection Tailored responses	Slow response times Lack of actionable guidance
Rule-based coach	Fast and structured interaction Convenient clickable response options	Lack of depth Constrained conversation Limited personalization

#### Aimi: Conversational Flexibility but Limited Actionability

Participants described Aimi as demonstrating contextual understanding and adaptive responsiveness, creating a fluid conversational experience that was relevant to their inputs. They appreciated the coach’s ability to maintain coherence even when conversations diverged from the initial direction. One participant noted:

Even when I side-tracked the answers, the coach will eventually connect back to the core response.

[P15, female]

Participants also highlighted that the sessions were personalized:

The coach did take my free text responses into account, eg, hiking, which made the conversation more relevant.

[P03, female]

The conversational flexibility fostered engagement, with one participant describing it as

by far the most intuitive and informative lifestyle coach of the three, I felt motivated to exercise after speaking with it.

[P13, male]

However, this conversational strength was undermined by excessive reflection and summarization. Participants consistently described this feature as robotic and frustrating, with the most frequent criticism being that the coach’s paraphrasing felt redundant. One participant explained:

“The coach repeated my sentences all the time to agree with me, it got kind of annoying after a while.”

[P04, male]

Another elaborated:

I understand that the coach was trying to repeat my response to make me feel heard and understood but sometimes the responses get too long and I feel that’s a bit redundant

[P05, female]

Despite Aimi’s ability to elicit motivation, the excessive questioning without concrete guidance limited its practical value. Participants were frustrated by the high question-to-suggestion ratio and the coach’s reluctance to provide direct advice. As one user described it:

Too many questions instead of really offering a suggestion. And when I asked the coach for a suggestion, it is still offering a suggestion as a question. The conversation becomes not so pleasing at the end

[P16, female]

Another participant expressed similar frustration:

The coach asked me questions about how I think something should help me, for the things I felt he should direct me. For example, how I think doing walk exercise will help me with my health issues. This should be instructed by the coach, not me.

[P17, male]

#### Novice Human Coach: Authentic Connection but Logistical Limitations

The novice human coach was perceived to be more emotionally attuned, and participants could sense that responses were tailored to their needs based on genuine human understanding. Participants consistently emphasized the authentic quality of the interaction and the coach’s commitment to exploring deeper meaning and individual context. One participant noted:

The coaching seemed very tailored, so everything we discussed seemed very relevant to the situation I described, ie, the agreement at the end also seemed like something that I could actually do.

[P03, female]

The human touch was also widely appreciated, with one participant describing the experience as

informative and felt like I was talking to a human, there was that personal touch.

[P13, male]

However, despite these relational strengths, slow response times significantly compromised the user experience. The latency problem was so severe that it offset the conversational advantages and created user frustration. One participant captured this tension:

I like this coach trying to understand why I want to make changes in my life and what this means to me. However, the responses were very slow and I lost interest in continuing this conversation.

[P05, female]

Another stated bluntly:

I am not really enjoying this coaching session because the delay was significant.

[P16, female]

Beyond the responsiveness issue, participants also noted that emotional support did not translate into tangible planning or concrete guidance. While the human coach provided some general tips, participants felt the coaching stopped short of actionable next steps. One user reflected:

The coach gave me reasonable tips for my first start at increasing my physical activity levels.

[P17, male]

but another expressed the limitation:

The answers given were merely words of encouragement. Although this can be motivational, providing actual suggestions might be better.

[P18, female]

#### Rule-Based CA: Efficient Delivery but Lacking Depth

The rule-based CA delivered rapid, structured interactions with clear clickable response options that many users appreciated for convenience. Participants valued the streamlined experience:

I enjoyed that the coach gave relevant options to reply to the questions.

[P08, female]

and

The option for clicking an answer is very convenient.

[P12, female]

The speed and simplicity were genuine strengths for users seeking quick information delivery.

However, this efficiency came at the cost of conversational depth and relational engagement. Participants consistently described the interaction as overly factual, constrained, and emotionally disengaged. The predefined answer options, while convenient, limited the direction and authenticity of the conversation. One participant explained:

The coach was more focused on general facts than going more into the detail on myself.

[P12, female]

while another emphasized the need for flexibility:

“I prefer two-way conversation that is more fluid and flexible.”

[P07, female]

The constraining nature of the interaction was particularly noted:

“It was very abrupt and I felt restricted with my answers

[P20, female]

Despite providing informative content, participants felt that the coach did not help translate this information into personalized action plans. One user reflected:

The coach did not really give me a plan to change my habits in the end. It only informed me about definitions etc.

[P04, male]

Another noted:

The coach provided some tips, but how I integrated that into my life and achieve my goal is missing.

[P05, female]

Additionally, a common criticism was that the coach’s predefined responses often felt assumptive and reduced trust:

There were set responses to choose from and without other questions it felt like the coach was assuming how I felt, making it feel less likely to trust this coach.

[P07, female]

## Discussion

### Principal Findings

The study provides foundational proof-of-concept evidence for the feasibility of using an LLM-based CA to deliver high-fidelity MI dialogue. The results establish the technical feasibility of Aimi, demonstrating that structured workflows can emulate the core processes and strategies of MI. As measured by the MISC-2 coding, the system achieved fidelity scores similar to those of a novice MI-trained human coach, suggesting that the MI Dialogue Engine functions as intended within the constraints set by the workflow (RQ1).

A key finding was the performance of the system relative to the rule-based CA: unconstrained by the rigid decision trees, Aimi could generate open-ended questions and complex reflections while engaging in nuanced, contextually relevant responses that aligned with MI principles. LLM-based CAs may therefore offer a viable approach for automating MI counseling tasks that were previously limited by the linguistic rigidity of rule-based systems.

No significant differences were observed in the user experience scores across the conditions (RQ2). However, descriptive feedback revealed subtle differences in perceived coaching interaction quality. Participants described Aimi as contextually relevant and responsive to their input, which made the interaction feel personalized, but the overuse of reflections eventually caused frustration. Furthermore, the inherent technical characteristics of each approach, such as the long latency of the novice human coach and the predefined answer options of the rule-based CA, distinctly shaped the user experience; the human coach was viewed as more emotionally authentic but slow to respond, whereas the rule-based CA was perceived as efficient but impersonal. Together, these findings highlight the importance of balancing naturalistic conversational flow and efficiency to deliver an authentic digital coaching experience.

### Comparison With Prior Work

#### Feasibility

Consistent with recent literature, our findings reinforce the growing evidence that artificial intelligence (AI)–driven MI coaching is feasible to implement and may enhance users’ motivation to change health behaviors. In a recent scoping review, Karve et al [41] summarized studies using AI for MI delivery, reporting that most interventions, whether rule-based or AI-based, demonstrated improvements in motivation, confidence, and readiness to change across domains such as physical activity [26,38], smoking cessation [23-25], alcohol and substance use [21,22,30,42], and general lifestyle [27,28,43]. Our study extends this literature by contextualizing the performance of a structured LLM-workflow–based CA alongside a novice MI-trained human coach and a rule-based CA, a gap not explicitly addressed in prior work.

#### Performance and Fidelity

Our findings show that participants rated Aimi as broadly equivalent to a novice MI-trained human coach across measures such as linguistic quality, dialogue relevance, and engagement, in line with recent reports that digital coaches and AI agents can provide relational support [44-46]. However, Aimi outperformed the human coach in terms of response latency (median 4.80, IQR 4.3-5.4 seconds vs median 38.09, IQR 18.1-75.73 seconds), with participants frequently reporting disengagement due to long delays in human responses. This observation underscores a key advantage of CAs in delivering scalable, on-demand intervention support unconstrained by human resource limitations and constrained primarily by the underlying LLM’s inference throughout, which is already capable of serving billions of prompts daily [47].

In terms of MI fidelity, Aimi achieved a median of 100% MI-consistent utterances. This aligns with other LLM-based MI systems that show MI consistency can approach, or even slightly surpass, human performance. For instance, Jörke et al [26] reported 93.3% MI-consistent utterances in a physical activity intervention, while Yang et al [43] found 96.6% MI-consistency using GPT-4o as an underlying LLM for transtheoretical behavioral change coaching. Furthermore, Aimi could also consistently evoke client change-talk. Given that eliciting change-talk is the core theoretical mechanism of MI, these results suggest that Aimi is capable of operationalizing MI.

However, such high fidelity scores also uncover an interesting “fidelity-engagement” paradox. Despite high expert-rated technical scores, feedback from participants suggested that Aimi’s frequent usage of reflections and summarizations felt excessive or robotic, which negatively affected their experience. Similar findings were reported by Steenstra et al [22], where the agent’s overreliance on complex reflections was perceived as redundant. Rather than running in tandem, our results highlight an important trade-off between therapeutic fidelity and authentic engagement. Furthermore, an overuse of reflections might also trigger the so-called “uncanny valley,” where excessive expressions of empathy appear performative rather than genuine [48]. Ultimately, this suggests that future LLM-based CAs must learn to balance technical accuracy with natural conversation rather than solely optimizing for strict protocol adherence.

#### Prompt Design and Intervention Architecture

Prompt design is increasingly recognized as a key strategy to improve the structure of coaching sessions in LLM-based MI delivery. Prior studies [22,24,27,30] have typically adopted single-prompt approaches, in which the LLM is instructed to act as an MI expert by specifying the desired persona, tone, and conversational structure. While straightforward, this approach is prone to model hallucinations and conversational drift as the session progresses [26,31]. In contrast, our study adopted a workflow-based prompt-chaining strategy, which decomposes lengthy and complex instructions into smaller, manageable tasks aligned with specific MI dialogue states. This method provides more reliable guidance and reduces the risk of drift throughout the conversation.

The workflow approach may also explain the system’s technical performance in eliciting change talk. By systematically monitoring progression through strict MI stage-based goal verification, the Stage Analyzer task ensured that Aimi refrained from offering advice too early or prematurely rushed to the planning stage. Ultimately, by structurally enforcing adherence to MI principles, the system potentially mirrored the behavior of a highly disciplined MI practitioner. Comparable strategies have emerged in the literature. For example, Jörke et al [26] implemented a prompt chain to guide physical activity coaching sessions across defined “dialogue states” (eg, motivation and goal setting), applying MI strategies conditioned on each state. Similarly, Meyer and Elsweiler [28] combined template-driven framing with natural language understanding for enhanced valence classification, generating free-text responses only during selected MI phases (eg, evoking). Together, these workflow-based and hybrid methods represent best current practices to balance flexibility, safety, and conversational coherence in LLM-mediated MI delivery.

#### User Expectations and Design Implications

A recurring theme from participant feedback was the expectation that the coach would provide immediate actionable advice. However, MI requires self-exploration, which is inherently effortful, requiring sustained cognitive engagement from users. User adoption and continued use depend heavily on perceived value and usability, and interactions that feel too effortful or misaligned with user expectations may lead to disengagement [49,50]. Conveying the purpose and demands of MI-oriented interactions may be a critical step when implementing digitally-delivered MI to ensure user expectations are aligned with MI and to prevent disengagement.

Within MI, effective behavior change also depends on the relational capacity of the coach. Specifically, MI theory distinguishes between two active ingredients that jointly facilitate behavior change: the technical component, encompassing specific counseling strategies and processes (eg, reflections, open questions, and elicitation of change talk), and the relational component—often referred to as the “spirit” of MI—reflecting empathy, collaboration, and the quality of the therapeutic relationship [9]. In traditional in-person MI sessions, these components are tightly interlinked. Skilled practitioners draw on nonverbal and paralinguistic cues, such as tone of voice, facial expressions, and body language, to balance technical delivery with relational responsiveness, tune in to emerging change talk, and adapt the conversation in real time. However, in a purely text-based format, the lack of access to such cues constrains the CA’s capacity to enact the more relational dimension of MI delivery. This limitation may help explain why some participants perceived Aimi as less useful or responsive, especially those seeking more immediate advice.

On the other hand, the relative strength of digital coaching agents may lie precisely in the technical aspects of MI. By applying MI-consistent strategies, LLM-based CAs may support users in clarifying their motivations and readiness for change, even in the absence of a fully realized relational component. From this perspective, MI CAs may function less as substitutes for human counseling but more as adjunctive or preparatory tools, providing an initial impetus for reflection that encourages users to seek further in-person support where the relational ingredient of behavior change can be more fully realized. Future digital coaching systems may therefore benefit from explicitly positioning LLM-based MI CAs as complementary supports that prioritize technical fidelity while facilitating onward engagement with blended human-delivered interventions.

Additionally, the effectiveness of this technical delivery is likely to be contingent on users’ readiness to engage in reflective change processes. Individuals in earlier stages of change (precontemplation or contemplation) may be more receptive to reflection-based MI strategies that build awareness and intrinsic motivation, whereas those in later stages (preparation, action, or maintenance) may benefit from directive MI-consistent techniques, such as goal setting, action planning, and strengthening commitment to change [51]. This suggests that LLM-based MI CAs should not be conceptualized as static implementations of MI, but as adaptive systems capable of modulating technical strategy in response to users’ stage of change. Such tailoring aligns with evidence from mobile health and digital MI research indicating that user engagement and satisfaction improve when interaction style is matched to readiness and individual needs [52].

Furthermore, this adaptive approach can be also extended to alleviate the “intention-action gap”—while MI is designed to strengthen a client’s intrinsic motivation and intention to change, the CA cannot inherently ensure real-world action. To bridge this, future LLM-based CAs could integrate with objective wearable data to monitor behavioral outcomes [53]. If the system detects that a user did not execute a planned behavior (eg, a scheduled walk), the CA could dynamically adjust its strategy, shifting from action-oriented planning back toward exploring ambivalence or rolling with resistance.

Finally, users in this study appreciated the speed, structure, and low-effort interaction of the rule-based coach but perceived it as too rigid, generic, and impersonal to support behavior change effectively. This highlights that rule-based and LLM-based approaches each offer distinct yet potentially complementary strengths in the delivery of MI. A promising future direction may lie in developing hybrid architectures [44,54,55] that leverage the reliability and efficiency of rule-based structures alongside the adaptive, context-aware capabilities of LLMs.

### Safety and Trust

LLM-based CAs present unique safety constraints, notably the well-known tendency for models to generate medically unsound or ungrounded responses [56]. Although a systematic clinical safety evaluation was outside the scope of this formative study, the off-topic detection node functions as a structural safeguard against scope-creep into clinical advice; no instances of unsafe advice were observed by the MI coder. A dedicated safety evaluation is planned for follow-up work.

Beyond unsafe responses, LLM-based CAs also pose an increased risk of anthropomorphism: the linguistic features that make MI effective—empathic reflections, collaborative stance, and conversational warmth—are known to prompt users to attribute human understanding, care, and therapeutic agency to dialogue systems [57]. This can potentially lead to misplaced trust, overdisclosure, and confusion about responsibility and expertise. Future work must explicitly address expectation management through transparent framing of the limits and nonhuman nature of LLM-based support.

### Limitations

The primary limitation of this study is that its clinical generalizability is constrained by the nature of the cohort and the research design. Specifically, the reliance on a small sample of young, tech-literate participants and a role-playing paradigm limits the study’s broader applicability. Furthermore, due to the logistical constraints of the human coach, true probabilistic randomization was also not feasible. Thus, we cannot rule out order or carryover effects as potential confounding variables, meaning our comparative results must be interpreted as preliminary trends rather than definitive causal findings. However, these choices were necessary to prioritize the feasibility and technical validation of the approach. The standardized baseline provided by the personas allowed for the isolation of system performance from confounding clinical variables. Future research is required to evaluate whether these findings translate to more diverse, less digitally proficient populations and to assess sustained behavioral impact in real-world clinical settings.

Second, the disparate technical characteristics of the three coaching approaches—most notably the varying response latencies and availability of predefined answer options—limited strict blinding. In this study, we observed that these “confounds” categorically shaped the user experience, with participants negatively perceiving the novice human coach’s slower responses and appreciating the rule-based CA’s relevant answer options. Since masking such characteristics is not feasible, future work must explicitly measure the extent to which participants identified the coaching condition and control for perceived scores appropriately.

Third, to assess fidelity to MI principles, the evaluation relied on ratings provided by a single expert MI coder. Given the reliance on a single coder, these results should be viewed as preliminary in nature. While this approach ensured consistency and depth of domain expertise, reliance on a single human evaluator represents a known limitation [58]. Future studies would benefit from incorporating multiple independent raters, interrater reliability assessments, and complementary automated evaluation methods to strengthen robustness and reproducibility.

Finally, there remains a lack of consensus regarding appropriate and standardized criteria for evaluating LLM-based CAs [59]. Our use of the Session Alliance Inventory [39]—originally validated for repeated human-to-human psychotherapy—in single 20-minute sessions represents an off-label application; ratings should therefore be interpreted as an indicator of immediate session-level rapport rather than therapeutic alliance in the validated sense. The development and adoption of shared evaluation frameworks would facilitate objective benchmarking and enable meaningful comparison across studies.

### Conclusion

This proof-of-concept study validates the feasibility of a structured LLM-workflow designed for MI. Results indicate that Aimi effectively maintained MI-fidelity at levels comparable to a novice human coach and directionally higher than the rule-based CA. It was also perceived as providing high linguistic quality and relevance similar to the other approaches, though an overuse of reflections and summarization were areas for improvement. Our study verifies the capacity of LLM-workflows to generate nuanced therapeutic dialogue, providing a technical baseline for future longitudinal studies on behavior impact.

## Supplementary material

10.2196/94036 Multimedia Appendix 1 Example of the intervention specification.

10.2196/94036 Multimedia Appendix 2 Prompts used in the Aimi large language model workflow.

10.2196/94036 Multimedia Appendix 3 Post session perceived user experience questionnaire.

10.2196/94036 Multimedia Appendix 4 Information sheet and consent form for participants.

## Abbreviations

AI: artificial intelligence
CA: conversational agent
LLM: large language model
MI: motivational interviewing
MISC-2: Manual for the Motivational Interviewing Skill Code Version 2.0
NCD: noncommunicable disease
OARS: open-ended questions, affirmations, reflective listening, and summarizing
RQ: research question

## References

[1] Brauer M, Roth GA, Aravkin AY (2024). Global burden and strength of evidence for 88 risk factors in 204 countries and 811 subnational locations, 1990–2021: a systematic analysis for the Global Burden of Disease Study 2021. The Lancet.

[2] Li J, Pandian V, Davidson PM, Song Y, Chen N, Fong DYT (2025). Burden and attributable risk factors of non-communicable diseases and subtypes in 204 countries and territories, 1990–2021: a systematic analysis for the global burden of disease study 2021. Int J Surg.

[3] Treciokiene I, Postma M, Nguyen T (2021). Healthcare professional-led interventions on lifestyle modifications for hypertensive patients – a systematic review and meta-analysis. BMC Fam Pract.

[4] Zhang X, Imperatore G, Thomas W (2017). Effect of lifestyle interventions on glucose regulation among adults without impaired glucose tolerance or diabetes: A systematic review and meta-analysis. Diabetes Res Clin Pract.

[5] Madigan CD, Graham HE, Sturgiss E (2022). Effectiveness of weight management interventions for adults delivered in primary care: systematic review and meta-analysis of randomised controlled trials. BMJ.

[6] Bellou V, Belbasis L, Tzoulaki I, Evangelou E (2018). Risk factors for type 2 diabetes mellitus: An exposure-wide umbrella review of meta-analyses. PLoS ONE.

[7] Tessier AJ, Wang F, Korat AA (2025). Optimal dietary patterns for healthy aging. Nat Med.

[8] Amiri S, Mahmood N, Javaid SF, Khan MA (2024). The Effect of Lifestyle Interventions on Anxiety, Depression and Stress: A Systematic Review and Meta-Analysis of Randomized Clinical Trials. Health Care (Don Mills).

[9] Miller WR, Rollnick S (2023). Motivational Interviewing: Helping People Change and Grow.

[10] Frost H, Campbell P, Maxwell M (2018). Effectiveness of Motivational Interviewing on adult behaviour change in health and social care settings: A systematic review of reviews. PLoS ONE.

[11] Zhu S, Sinha D, Kirk M (2024). Effectiveness of behavioural interventions with motivational interviewing on physical activity outcomes in adults: systematic review and meta-analysis. BMJ.

[12] Weisner C, Satre DD (2016). A key challenge for motivational interviewing: training in clinical practice. Addiction.

[13] Milne-Ives M, de Cock C, Lim E (2020). The Effectiveness of Artificial Intelligence Conversational Agents in Health Care: Systematic Review. J Med Internet Res.

[14] He Y, Yang L, Qian C (2023). Conversational Agent Interventions for Mental Health Problems: Systematic Review and Meta-analysis of Randomized Controlled Trials. J Med Internet Res.

[15] Kowatsch T, Schachner T, Harperink S (2021). Conversational Agents as Mediating Social Actors in Chronic Disease Management Involving Health Care Professionals, Patients, and Family Members: Multisite Single-Arm Feasibility Study. J Med Internet Res.

[16] Ollier J, Suryapalli P, Fleisch E (2023). Can digital health researchers make a difference during the pandemic? Results of the single-arm, chatbot-led Elena+: Care for COVID-19 interventional study. Front Public Health.

[17] Castro O, Mair JL, Salamanca-Sanabria A (2023). Development of “LvL UP 1.0”: a smartphone-based, conversational agent-delivered holistic lifestyle intervention for the prevention of non-communicable diseases and common mental disorders. Front Digit Health.

[18] Mair JL, Jabir AI, Salamanca-Sanabria A (2025). Feasibility of the LvL UP digital lifestyle coaching intervention designed to prevent non-communicable diseases and common mental disorders. Sci Rep.

[19] Bickmore TW, Schulman D, Sidner CL (2011). A reusable framework for health counseling dialogue systems based on a behavioral medicine ontology. J Biomed Inform.

[20] Martinengo L, Lin X, Jabir AI (2023). Conversational Agents in Health Care: Expert Interviews to Inform the Definition, Classification, and Conceptual Framework. J Med Internet Res.

[21] Yeo YH, Clark A, Mehra M (2024). The feasibility and usability of an artificial intelligence-enabled conversational agent in virtual reality for patients with alcohol-associated cirrhosis: A multi-methods study. JMedXR.

[22] Steenstra I, Nouraei F, Arjmand M, Bickmore T (2024). Virtual agents for alcohol use counseling: exploring LLM-powered motivational interviewing.

[23] Brown A, Kumar AT, Melamed O (2023). A Motivational Interviewing Chatbot With Generative Reflections for Increasing Readiness to Quit Smoking: Iterative Development Study. JMIR Ment Health.

[24] Kumar AT, Wang C, Dong A, Rose J (2024). Generation of Backward-Looking Complex Reflections for a Motivational Interviewing–Based Smoking Cessation Chatbot Using GPT-4: Algorithm Development and Validation. JMIR Ment Health.

[25] Mahmood Z, Ali S, Zhu J, Che W, Nabende J, Shutova E, Pilehvar MT (2025). A fully generative motivational interviewing counsellor chatbot for moving smokers towards the decision to quit. https://aclanthology.org/2025.findings-acl.

[26] Jörke M, Sapkota S, Warkenthien L (2025). GPTCoach: towards LLM-based physical activity coaching.

[27] Meywirth S, Mandviwalla M, Söllner M, Tuunanen T (2024). Design Science Research for a Resilient Future.

[28] Meyer S, Elsweiler D (2025). LLM-based conversational agents for behaviour change support: A randomised controlled trial examining efficacy, safety, and the role of user behaviour. Int J Hum Comput Stud.

[29] Liu R, Geng J, Peterson J, Sucholutsky I, Griffiths TL (2025). Large language models assume people are more rational than we really are. https://openreview.net/forum?id=dAeET8gxqg.

[30] Herbert D, Westendorf J, Farmer M, Reeder B (2025). Generative AI-Derived Information About Opioid Use Disorder Treatment During Pregnancy: An Exploratory Evaluation of GPT-4’s Steerability for Provision of Trustworthy Person-Centered Information. J Stud Alcohol Drugs.

[31] Laban P, Hayashi H, Zhou Y, Neville J (2025). LLMs get lost in multi-turn conversation. arXiv.

[32] Goldberg CB, Adams L, Blumenthal D (2024). To do no harm — and the most good — with AI in health care. Nat Med.

[33] Introducing GPT-4.5. OpenAI.

[34] Touvron H, Lavril T, Izacard G (2023). LLaMA: open and efﬁcient foundation language models. arXiv.

[35] Sahoo P, Singh AK, Saha S, Jain V, Mondal S, Chadha A (2025). A systematic survey of prompt engineering in large language models: techniques and applications. arXiv.

[36] microsoft/botframework-sdk: bot framework provides the most comprehensive experience for building conversation applications. GitHub.

[37] de Jonge JM, Schippers GM, Schaap CPDR (2005). The Motivational Interviewing Skill Code: Reliability and a Critical Appraisal. Behav Cogn Psychother.

[38] Sun X, de Wit J, Li Z, Pei J, El Ali A, Bosch JA (2025). Script-Strategy Aligned Generation: Aligning LLMs with Expert-Crafted Dialogue Scripts and Therapeutic Strategies for Psychotherapy. Proc ACM Hum-Comput Interact.

[39] Falkenström F, Hatcher RL, Skjulsvik T, Larsson MH, Holmqvist R (2015). Development and validation of a 6-item working alliance questionnaire for repeated administrations during psychotherapy. Psychol Assess.

[40] Sim J (2019). Should treatment effects be estimated in pilot and feasibility studies?. Pilot Feasibility Stud.

[41] Karve Z, Calpey J, Machado C, Knecht M, Mejia MC (2025). New Doc on the Block: Scoping Review of AI Systems Delivering Motivational Interviewing for Health Behavior Change. J Med Internet Res.

[42] Suffoletto B, Clark DB, Lee C (2025). Development and preliminary testing of a secure large language model-based chatbot for brief alcohol counseling in young adults. Drug Alcohol Depend.

[43] Yang Y, Achananuparp P, Huang H, Che W, Nabende J, Shutova E, Pilehvar MT (2025). CAMI: a counselor agent supporting motivational interviewing through state inference and topic exploration.

[44] Oh YJ, Liang KH, Kim DD (2025). Enhancing physical activity through a relational artificial intelligence chatbot: A feasibility and usability study. Digit Health.

[45] Basar E, Hendrickx I, Krahmer E, Bruijn GJ, Bosse T, Soni N, Flek L, Sharma A, Yang D, Hooker S, Schwartz HA (2024). To What Extent Are Large Language Models Capable of Generating Substantial Reflections for Motivational Interviewing Counseling Chatbots? A Human Evaluation.

[46] Nurmi J, Knittle K, Ginchev T (2020). Engaging Users in the Behavior Change Process With Digitalized Motivational Interviewing and Gamification: Development and Feasibility Testing of the Precious App. JMIR Mhealth Uhealth.

[47] Roth E (2025). OpenAI says ChatGPT users send over 2.5 billion prompts every day. The Verge.

[48] Seitz L (2024). Artificial empathy in healthcare chatbots: Does it feel authentic?. Comput Hum Behav Artif Hum.

[49] Mair JL, Hashim J, Thai L (2025). Understanding and overcoming barriers to digital health adoption: a patient and public involvement study. Transl Behav Med.

[50] Savir T, Baumel A (2025). Capacity to Invest Effort as a Predictor of Preference for Digital Mental Health Interventions Over Psychotherapy: Cross-Sectional Study Using an Ecological Digital Screening Tool. J Med Internet Res.

[51] DiClemente CC, Graydon MM, Hamilton K, Cameron LD, Hagger MS, Hankonen N, Lintunen T (2020). The Handbook of Behavior.

[52] Yoon S, Tang H, Tan CM, Phang JK, Kwan YH, Low LL (2024). Acceptability of Mobile App–Based Motivational Interviewing and Preferences for App Features to Support Self-Management in Patients With Type 2 Diabetes: Qualitative Study. JMIR Diabetes.

[53] Heydari AA, Gu K, Srinivas V (2025). The anatomy of a personal health agent. arXiv.

[54] Moore R, Al-Tamimi AK, Freeman E (2024). Investigating the Potential of a Conversational Agent (Phyllis) to Support Adolescent Health and Overcome Barriers to Physical Activity: Co-Design Study. JMIR Form Res.

[55] Vinay R, Uetova E, Tommila NC, Biller-Andorno N, Kowatsch T (2025). A Hybrid Rule- and Large Language Model-Based Embodied Voice Assistant (GRACE) for Cognitive Stimulation in Older Adults: Usability Study Assessing Technical Feasibility, Technology Acceptance, and Working Alliance. JMIR Aging.

[56] Draelos RL, Afreen S, Blasko B (2026). Large language models provide unsafe answers to patient-posed medical questions. npj Digit Med.

[57] Abercrombie G, Curry A, Dinkar T, Rieser V, Talat Z, Bouamor H, Pino J, Bali K (2023). Mirages. on anthropomorphism in dialogue systems.

[58] Cofie N, Braund H, Dalgarno N (2022). Eight ways to get a grip on intercoder reliability using qualitative-based measures. Can Med Educ J.

[59] Abbasian M, Khatibi E, Azimi I (2024). Foundation metrics for evaluating effectiveness of healthcare conversations powered by generative AI. npj Digit Med.

