# Supplementary material for: Structured Large Language Model Workflows for Motivational Interviewing in Health Behavior Change: Proof-of-Concept Study
Source: JMIR Form Res. 2026 Jul 6;10:e94036. doi: 10.2196/94036 (PMC13336328; doi:10.2196/94036)
Supplement: Multimedia Appendix 1 [file formative-v10-e94036-s001.docx]

## Example of the Intervention Specification

| 1. Intervention Summary  This intervention is designed to promote behaviour change in individuals with low to moderate physical activity levels by using Motivational Interviewing (MI) principles. The intervention focuses on increasing self-efficacy, addressing barriers to physical activity, and supporting individuals in adopting and maintaining an active lifestyle. Through personalized goal-setting, behavioural strategies, and structured progress tracking, the intervention provides a tailored approach to help individuals gradually increase their physical activity in a sustainable way.  2. Target Population   - Adults (18-65 years) with low to moderate levels of physical activity - Individuals who are not meeting current physical activity guidelines (less than 150 minutes of moderate-intensity exercise per week) - People who express readiness for change but may struggle with motivation or adherence   3. Target Behaviour   - Increase in overall physical activity levels - Adoption of regular moderate-intensity physical activity, such as walking, cycling, or structured exercise sessions - Reduction in sedentary behaviour through incremental lifestyle changes   4. Guidelines/Prescription  Initial Assessment: The chatbot will assess users' current activity levels, motivation, and potential barriers.  Stratification Criteria:   - Low Activity (<75 min/week): Focus on building confidence, addressing barriers, and introducing light activity (e.g., 10-minute daily walks). - Moderate Activity (75-149 min/week): Encourage progression to meet or exceed the 150-minute guideline with structured goal setting.  1. Behavioural Strategies:  - Personalized goal setting (e.g., SMART goals) - Activity tracking and progress feedback - Social support integration (e.g., connecting with peers or family members) - Reinforcement through positive affirmations and rewards  1. Follow-up and Adaptation:   The chatbot will regularly check in to assess progress, provide encouragement, and adjust recommendations as needed based on user feedback. |
| --- |

## Post Session Surveys

**Session Alliance Inventory (SAI)**

Six-point Likert scale [0-5]: [Not at all, A little, Moderately, Quite a bit, Very much, Completely]

- The coach and I were working towards mutually agreed upon goals.
- I felt that the coach appreciated me.
- The coach and I respected each other.
- We were in agreement on what is important for me to work on.
- I felt that the coach cared about me even if I had done things that they did not approve of.
- I believe the way we were working with my problem(s) was correct.

**Dialogue Relevance**

Five-point Likert scale [0-4]: [Strongly Disagree, Disagree, Neutral, Agree, Strongly Agree]

- The coach asked an appropriate amount of questions.
- The coach stayed on the specific topic of the conversation.
- The conversation was relevant.

**Linguistic Quality**

Five-point Likert scale [0-4]: [Strongly Disagree, Disagree, Neutral, Agree, Strongly Agree]

- The conversation was fluent.
- The conversation was natural.
- The conversation was coherent.

**Usability**

Five-point Likert scale [0-4]: [Strongly Disagree, Disagree, Neutral, Agree, Strongly Agree]

- Communicating with the coach was clear.
- The coach’s responses were easy to understand.
- The coach could handle situations in which the line of conversation was not clear.
- I feel like the coach’s responses were accurate.

**Empathy**

Five-point Likert scale [0-4]: [Strongly Disagree, Disagree, Neutral, Agree, Strongly Agree]

- The coach seemed to know how I was feeling.
- The coach seemed to understand me.

**Engagement**

Five-point Likert scale [0-4]: [Strongly Disagree, Disagree, Neutral, Agree, Strongly Agree]

- I enjoyed talking to the coach.

**Motivation**

Five-point Likert scale [0-4]: [Strongly Disagree, Disagree, Neutral, Agree, Strongly Agree]

- I feel motivated to make changes in my physical activity behaviour after talking with the coach.
- I feel like the coach understood what I wanted and can help me achieve my goal.
- The coach helped me talk about changing my behaviour.
- The coach helped me feel hopeful about changing my behaviour.

**Open feedback**

Free text

- What have you enjoyed most or least about this coaching session?
- What could be improved about this coaching session?

## Expert-rated MI Fidelity Results

| **Summary Scores** | **Conditions** | **Mean (SD)** | ***P*-value** |
| --- | --- | --- | --- |
| Ratio reflections/questions | Aimi vs. Human | 0.78 (0.26) vs. 0.64 (0.30) | .831 |
|  | Aimi vs. Rule-based | 0.78 (0.26) vs. 0.33 (0.23) | .003** |
|  | Human vs. Rule-based | 0.64 (0.30) vs. 0.33 (0.23) | .003** |
| Open questions (%) | Aimi vs. Human | 86.68 (12.64) vs. 64.09 (14.53) | .017* |
|  | Aimi vs. Rule-based | 86.68 (12.64) vs. 50.66 (18.03) | .001** |
|  | Human vs. Rule-based | 64.09 (14.53) vs. 50.66 (18.03) | .314 |
| Complex reflections (%) | Aimi vs. Human | 64.33 (20.58) vs. 48.85 (24.32) | .749 |
|  | Aimi vs. Rule-based | 64.33 (20.58) vs. 25.00 (30.92) | .030* |
|  | Human vs. Rule-based | 48.85 (24.32) vs. 25.00 (30.92) | .328 |
| MI consistent (%) | Aimi vs. Human | 98.13 (2.37) vs. 97.74 (3.26) | .100 |
|  | Aimi vs. Rule-based | 98.13 (2.37) vs. 99.72 (1.18) | .328 |
|  | Human vs. Rule-based | 97.74 (3.26) vs. 99.72 (1.18) | .433 |
| Client change talk (%) | Aimi vs. Human | 91.06 (9.05) vs. 71.72 (13.33) | .001** |
|  | Aimi vs. Rule-based | 91.06 (9.05) vs. 71.16 (16.15) | .038* |
|  | Human vs. Rule-based | 71.72 (13.33) vs. 71.16 (16.15) | .100 |
| **Global Scores** | **Conditions** | **Mean (SD)** | ***P*-value** |
| Overall Coach Rating | Aimi vs. Human | 4.11 (0.58) vs. 4.44 (0.51) | .749 |
|  | Aimi vs. Rule-based | 4.11 (0.58) vs. 3.50 (0.51) | .113 |
|  | Human vs. Rule-based | 4.44 (0.51) vs. 3.50 (0.51) | .012* |
| Acceptance | Aimi vs. Human | 5.83 (0.38) vs. 5.61 (0.53) | .100 |
|  | Aimi vs. Rule-based | 5.83 (0.38) vs. 5.25 (0.43) | .024* |
|  | Human vs. Rule-based | 5.61 (0.53) vs. 5.25 (0.43) | .741 |
| Empathy | Aimi vs. Human | 5.11 (0.58) vs. 5.03 (0.63) | .100 |
|  | Aimi vs. Rule-based | 5.11 (0.58) vs. 4.39 (0.50) | .061 |
|  | Human vs. Rule-based | 5.03 (0.63) vs. 4.39 (0.50) | .072 |
| MI spirit | Aimi vs. Human | 5.22 (0.73) vs. 4.92 (0.65) | .100 |
|  | Aimi vs. Rule-based | 5.22 (0.73) vs. 4.47 (0.85) | .235 |
|  | Human vs. Rule-based | 4.92 (0.65) vs. 4.47 (0.85) | .569 |
| Client self-exploration | Aimi vs. Human | 3.72 (0.49) vs. 3.81 (0.57) | .100 |
|  | Aimi vs. Rule-based | 3.72 (0.49) vs. 3.17 (0.49) | .113 |
|  | Human vs. Rule-based | 3.81 (0.57) vs. 3.17 (0.49) | .042* |

Table S1 Wilcoxon signed-rank tests comparing MI fidelity of coaches. (***P < .001, **P < .01, *P < .05).
