# Supplementary material for: Structured Large Language Model Workflows for Motivational Interviewing in Health Behavior Change: Proof-of-Concept Study
Source: JMIR Form Res. 2026 Jul 6;10:e94036. doi: 10.2196/94036 (PMC13336328; doi:10.2196/94036)
Supplement: Multimedia Appendix 3 [file formative-v10-e94036-s003.docx]

## Post Session Surveys

**Session Alliance Inventory (SAI)**

Six-point Likert scale [0-5]: [Not at all, A little, Moderately, Quite a bit, Very much, Completely]

- The coach and I were working towards mutually agreed upon goals.
- I felt that the coach appreciated me.
- The coach and I respected each other.
- We were in agreement on what is important for me to work on.
- I felt that the coach cared about me even if I had done things that they did not approve of.
- I believe the way we were working with my problem(s) was correct.

**Dialogue Relevance**

Five-point Likert scale [0-4]: [Strongly Disagree, Disagree, Neutral, Agree, Strongly Agree]

- The coach asked an appropriate amount of questions.
- The coach stayed on the specific topic of the conversation.
- The conversation was relevant.

**Linguistic Quality**

Five-point Likert scale [0-4]: [Strongly Disagree, Disagree, Neutral, Agree, Strongly Agree]

- The conversation was fluent.
- The conversation was natural.
- The conversation was coherent.

**Usability**

Five-point Likert scale [0-4]: [Strongly Disagree, Disagree, Neutral, Agree, Strongly Agree]

- Communicating with the coach was clear.
- The coach’s responses were easy to understand.
- The coach could handle situations in which the line of conversation was not clear.
- I feel like the coach’s responses were accurate.

**Empathy**

Five-point Likert scale [0-4]: [Strongly Disagree, Disagree, Neutral, Agree, Strongly Agree]

- The coach seemed to know how I was feeling.
- The coach seemed to understand me.

**Engagement**

Five-point Likert scale [0-4]: [Strongly Disagree, Disagree, Neutral, Agree, Strongly Agree]

- I enjoyed talking to the coach.

**Motivation**

Five-point Likert scale [0-4]: [Strongly Disagree, Disagree, Neutral, Agree, Strongly Agree]

- I feel motivated to make changes in my physical activity behaviour after talking with the coach.
- I feel like the coach understood what I wanted and can help me achieve my goal.
- The coach helped me talk about changing my behaviour.
- The coach helped me feel hopeful about changing my behaviour.

**Open feedback**

Free text

- What have you enjoyed most or least about this coaching session?
- What could be improved about this coaching session?
