# Supplementary material for: Structured Large Language Model Workflows for Motivational Interviewing in Health Behavior Change: Proof-of-Concept Study
Source: JMIR Form Res. 2026 Jul 6;10:e94036. doi: 10.2196/94036 (PMC13336328; doi:10.2196/94036)
Supplement: Multimedia Appendix 4 [file formative-v10-e94036-s004.pdf]

(SEC) SINGAPORE-ETH CENTRE (FHT) FUTURE HEALTH TECHNOLOGIES NUS National University of Singapore

# Information sheet for participants

**NUS-IRB Reference Code:** NUS-IRB-2025-98

1. **Protocol title**

   Using AI to deliver motivational interviewing support

2. **Principal Investigator and co-investigator(s):**


Principal Investigators:
Jacqueline Mair, Future Health Technologies, Singapore-ETH Centre

Co-Investigators:
Akshaye Shenoi Singapore-ETH Centre
Ahmad Jabir; Singapore ETH-Centre
Tianze Li; Singapore ETH-Centre
Oscar Castro; Singapore ETH-Centre
Amanda Pitkethly; Edinburgh Napier University
Jumana Hashim; National University of Singapore
Tai E Shyong; National University of Singapore


3. **What is the purpose of this research?**

   Our team has developed a new large language model (LLM)-based motivational interviewing conversational agent (or chatbot) designed to help people overcome barriers to lifestyle change. The aim of this study is to evaluate users' experience with different types of coaches: (1) an LLM-based chatbot, (2) a rule-based chatbot and (3) a human. Additionally, an expert will evaluate how closely each coaching type adheres to the principles of motivational interviewing.

4. **Who can participate in the research? What is the expected duration of my participation? What is the duration of this research?**

   Inclusion criteria are as follows:

   a) Aged 21-59 years
   b) Fluent in English
   c) Apparently healthy
   d) Ability to independently provide informed consent

   This study will run for approximately 1 month, but the duration of your participation will only be around 2 hours.

5. **What is the approximate number of research participants involved?**
   We aim to recruit 30 participants in total.

6. **What will be done if I take part in this research study?**
   If you take part in this study, you will be asked to attend one in-person session, along with up to 2 other people, held at the Singapore-ETH Centre, CREATE Tower, 1 Create Way, Singapore.

   Before you take part in the session you will be asked to fill out a short survey to check your eligibility for the study. You will then be contacted to schedule a convenient date and time to join a testing session in-person at the CREATE Campus.

   On the day of your scheduled session, you will be asked to read the study information sheet and provide your written consent to participate. A facilitator will then explain the purpose of the study and offer you an opportunity to ask any questions. You will be provided with either a laptop or iPad with keyboard and given a briefing leaflet that outlines your study ID and the steps you need to follow.

   You will be asked to complete three web-based coaching sessions (in a particular order) on the topic of physical activity. The coaching sessions are simulations of a real motivational interviewing session. When engaging with the coach you should assume a persona of a person who is struggling to engage in physical activity.

   Each session should take approximately 20 minutes to complete. On completion of each coaching session, you will fill out a short survey to provide your feedback. The entire session will last 90 - 120 minutes.

7. **How will my privacy and the confidentiality of my research records be protected?**
   Only the principal investigator and named members of the research team will have access to your personal data (e.g. names and contact information,) and this will not be released to any other person. Personal data will never be used in a publication or presentation. All identifiable research data will be coded (i.e. only identified with a code number) at the earliest possible stage of the research. Personal data will be discarded upon completion of the study.

   All data collected will be kept in accordance to the University's Research Data Management Policy. Research data used in any publication will be kept for a minimum of 10 years before being discarded.

8. **What are the possible discomforts and risks for participants?**
   There are no expected risks of taking part in this study.

9. **What is the compensation for any injury?**
   We do not expect you to be injured during the course of this project. If you follow the directions of the PI in charge of this research study and you are injured, the NUS will pay the medical expenses for the treatment of that injury. By giving

your consent, you will not waive any of your legal rights or release the parties involved in this study from liability for negligence.

10. **Will there be reimbursement for participation?**
If you take part in this study, you will receive a voucher to the value of S$30.00 for a reputable retailer as a thank you for your time and contribution.

If you take part in this study, you will have to pay for the following: transportation costs to and from the study location.

11. **What are the possible benefits to me and to others?**
There is no direct benefit to you by participating in this research study. The knowledge gained will shape the development of a new digital health coaching intervention aimed at supporting people to lead a healthy lifestyle. Your will be contributing towards the advancement of research in this area.

12. **Can I refuse to participate in this research?**
Yes, you can. Your decision to participate in this research study is voluntary and completely up to you. You can also withdraw from the research at any time without giving any reasons, by informing the principal investigator. Any data we collected from you will be discarded.

13. **Whom should I call if I have any questions or problems?**
Please contact the Principal Investigator, Dr Jacqueline Mair at **email** **Jacqueline.mair@sec.ethz.ch** for all research-related matters and in the event of research-related injuries.

For an independent opinion specifically regarding the rights and welfare of research participants, you may contact a staff member of the National University of Singapore Institutional Review Board at telephone (+65) 6516 1234 [Mondays to Thursdays from 8.30am to 6pm, and Fridays from 8.30am to 5.30pm, except public holidays] or email at irb@nus.edu.sg.

## Consent form

**Protocol title:**
Using AI to deliver motivational interviewing support

**Principal Investigator with the contact number and organization:**
Dr Jacqueline Mair; Singapore-ETH Centre; +65 88664980

I hereby acknowledge that:

1. I have agreed to take part in the above research.

2. I have received a copy of this information sheet that explains the use of my data in this research. I understand its contents and agree to donate my data for the use of this research.

3. I can withdraw from the research at any point of time by informing the Principal Investigator and all my data will be discarded.

4. I will not have any financial benefits that result from the commercial development of this research.

5. I *agree / do not agree** to be re-contacted for future related studies. I understand that future studies will be subject to an Institutional Review Board's approval.

6. I *agree / do not agree** to the audio-recording or video-recording of my participation in the research. I understand that although my name will be not associated with the video-recordings used in publication/presentation, I may still be identified.

**please delete as appropriate*

______________________________ ___________

Name and Signature (Participant) Date

______________________________ ___________

Name and Signature (Researcher) Date
